# Supplementary material for: Chemically modified graphene films with tunable negative Poisson’s ratios
Source: Nat Commun. 2019 Jun 4;10:2446. doi: 10.1038/s41467-019-10361-3 (PMC6547682; doi:10.1038/s41467-019-10361-3)
Supplement: Supplementary file 1 — Supplementary Information [file 41467_2019_10361_MOESM1_ESM.pdf]

Supplementary Information for

Chemically Modified Graphene Films with Tunable Negative Poisson's Ratios

Yeye Wen,[1,*] Enlai Gao,[2,*] Zhenxing Hu,[3,*] Tingge Xu,[3] Hongbing Lu,[3] Zhiping Xu,[4] and Chun Li[1]

[1]Department of Chemistry, MOE Key Laboratory of Bioorganic Phosphorus Chemistry & Chemical Biology, Tsinghua University, Beijing 100084, China

[2]Department of Engineering Mechanics, School of Civil Engineering, Wuhan University, Wuhan, Hubei 430072, China

[3]Department of Mechanical Engineering, the University of Texas at Dallas, 800 W. Campbell Rd. Richardson, TX, 75080, USA

[4]Applied Mechanics Laboratory, Department of Engineering Mechanics and Center for Nano and Micro Mechanics, Tsinghua University, Beijing 100084, China

[*]These authors contributed equally to this work.

Corresponding authors. E-mails: chunli@mail.tsinghua.edu.cn (C.L.), xuzp@tsinghua.edu.cn (Z.X.).

This Supplementary Information Material contains

- Supplementary Notes on *preparation of GO Dispersions*, *structural characterization of GO sheets*, *mechanical characterization*, and *theoretical model*.
- Supplementary Figures 1-9

Supplementary Note 1 – Preparation of GO Dispersions

GO_0 was synthesized by a modified Hummers method from natural graphite at a relatively low oxidation temperature, 0 ^{o}C. Graphite powder (325 mesh, 3.0 g) was dispersed in a concentrated sulfuric acid (70 mL) under stirring with a constant speed of 350 rpm at 0 °C. After stirring for 0.5 h, potassium permanganate (9 g) was added into flask slowly over a period of 2 h. The reaction mixture was kept stirring for additional 8 h at 0 °C. Then, deionized water (150 mL) was slowly added to the reaction mixture over a period of 6 h by a peristaltic pump. All the reaction processes were strictly controlled at 0 °C. The final reaction mixture was poured into ice-water mixture (1000 mL) to terminate the reaction, then hydrogen peroxide (30% aqueous solution) was added into the mixture under stirring until no gas escaped from the dispersion. The dispersion was filtrated and the filter cake was washed using diluted (3.7%) hydrochloric acid aqueous solution to remove the metal ions. The resultant slurry was then dispersed in distilled water under mild magnetic stirring to form a GO suspension. The GO suspension was purified by dialysis for two weeks. After that, it was centrifuged repeatedly at 3000 rpm to remove non-exfoliated aggregates. Finally, the GO suspension was concentrated by centrifugation at 10,000 rpm for 1 h. The concentration was evaluated to be at 7.40 mg mL^{-1} by weighting the dried solid from 1 mL concentrated GO dispersion.

GO_{35} was prepared by the oxidation of graphite powder (325 mesh, 3.0 g) following a modified Hummers' method. Briefly, graphite was added into the concentrated sulfuric acid (70 mL) under stirring at room temperature. Sodium nitrate (1.5 g) was added into the mixture and the reaction mixture was then cooled to 0 °C, followed by slowly adding potassium permanganate (9.0 g) under vigorous agitation to keep the temperature of the suspension lower than 20 °C. Then the reaction mixture was transferred to a 35-40 °C water bath for 30 min, successively, deionized water (150

mL) was added and reaction mixture was stirred for another 15 min at 90-95 °C. Deionized water (500 mL) was added into reaction mixture followed by the addition of hydrogen peroxide (30% aqueous solution, 20 mL). Finally, the dispersion was purified following the procedures for GO_0 preparation. The concentration of GO_{35} was evaluated to be at 7 mg mL^{-1}.

Supplementary Note 2 – Structural Characterization of GO Sheets

Scanning electron microscopy (SEM) images and the corresponding size distribution histograms demonstrate that GO_0 and GO_{35} sheets have an average lateral size of 12.5 and 13.5 μm, respectively (Figs. S1a, S1b, S1d, S1e). XPS surveys indicate that GO_0 sheets have a higher C/O ratio (2.29) than GO_{35} sheets (1.84), and the C 1s XPS peaks can be deconvoluted into five characteristic peaks, C=C (~284.7 eV), C−C (285.5 eV), C−OH/C−O−C (~286.7 eV), C=O (~287.6 eV) and HO−C=O (~289.2 eV) (Fig. S1c). The Raman spectra (Fig. S1f) display typical D- (1340 cm^{-1}) and G-band (1580 cm^{-1}) of GO, giving an I_D/I_G of 1.03 for GO_0 and 0.96 for GO_{35}, respectively. These results indicate that GO_0 and GO_{35} possess different chemical structures. GO_0 sheets prepared at low oxidation temperature of 0 ^oC have lower degree of oxidation, less defects and larger graphitic domains compared to GO_{35} sheets prepared by the conventional Hummer's method at the oxidation temperature of 35 °C.

Supplementary Note 3 – Mechanical Characterization

GO_{35} films show uniaxial tensile strength of 110 ± 22 MPa and strain to failure of 1.75 ± 0.26 %. Comparing with GO_{35} films, GO_0 films exhibit improved mechanical properties with tensile strength of 252 ± 16 MPa and strain to failure of 3.45 ± 0.51 %, with a benefit from the structural integrity of GO_0 precursors. Post-reduction of GO_0 films gave robust rGO_0 film with higher tensile strength (402 ± 18 MPa) and strain to failure (5.24 ± 0.12 %).

Supplementary Note 4 – Theoretical Model

Based on SEM and optical images of the cross-section and surface topography of CMG films (Figs. 4a-b), we constructed a schematic illustration of the microstructures for monolithic CMG films (Fig. 4c), which consists of an inter-connected network of CPLs (CMG multilayers with typical thickness of ~10-40 nm). With the structural features included in this model, one can establish quantitative understandings of the NPR effect. We consider a representative volume element (RVE) to capture the wavy texture of the inter-connected network of CPLs (Fig. 4d), where the orthotropic (solid, dash) sticks represent the disordered stacks, wrinkles, delamination of CPLs. The angle θ formed between the sticks and the basal plane of CMG films measures the amplitude of out-of-plane corrugation in CPLs, which can be related to the polarized Raman measurement of the alignment angle α. To correlate the value of NPRs with the alignment angle, the following assumption and analysis are made. Under a stretch force F on the CPL, the out-of-plane corrugation induced by disordered stacks, wrinkles and delamination will be reduced, resulting in extension in both tensile and lateral directions. In the tensile direction, this extension can be measured by displacements Δ_1 and Δ_2, that are

$$\Delta_1 = F\sin\theta s^3/3D,\ \Delta_2 = F\cos\theta s/K \tag{S1}$$

where D and K are the effective bending and tensile stiffness of the corrugated CPLs that are represented by sticks with a length of s.

The in-plane deformation of a RVE can be measured as

$$\Delta l = \Delta_1\sin\theta + \Delta_2\cos\theta,\ \Delta h = \Delta_2\sin\theta - \Delta_1\cos\theta \tag{S2}$$

As $w^2 = s^2 - h^2$, the change in the RVE width is $\Delta w = -h\Delta h/w = -\Delta h\tan\theta$, and thus the Poisson's ratio is

$$\nu_{YX} = -\frac{\Delta w / w}{\Delta l / l} = \frac{(1 - \frac{s^2 K}{3D}) \tan^2 \theta}{1 + \frac{s^2 K}{3D} \tan^2 \theta} \tag{S3}$$

by assuming $w = l$ with the assumption of in-plane isotropy in the CMG films. We also assume that $\tan\alpha$ is a linearly function of $\tan\theta$ with a pre-factor N ($\tan\theta = h/l = N\tan\alpha$).

Finally, denote $s^2K/3D$ as M, the Poisson's ratio can be expressed as

$$\nu_{YX} = \frac{(1 - M) N^2 \tan^2 \alpha}{1 + M N^2 \tan^2 \alpha} \tag{S4}$$

which is used to plot Fig. 4d.

Supplementary Figures and Captions

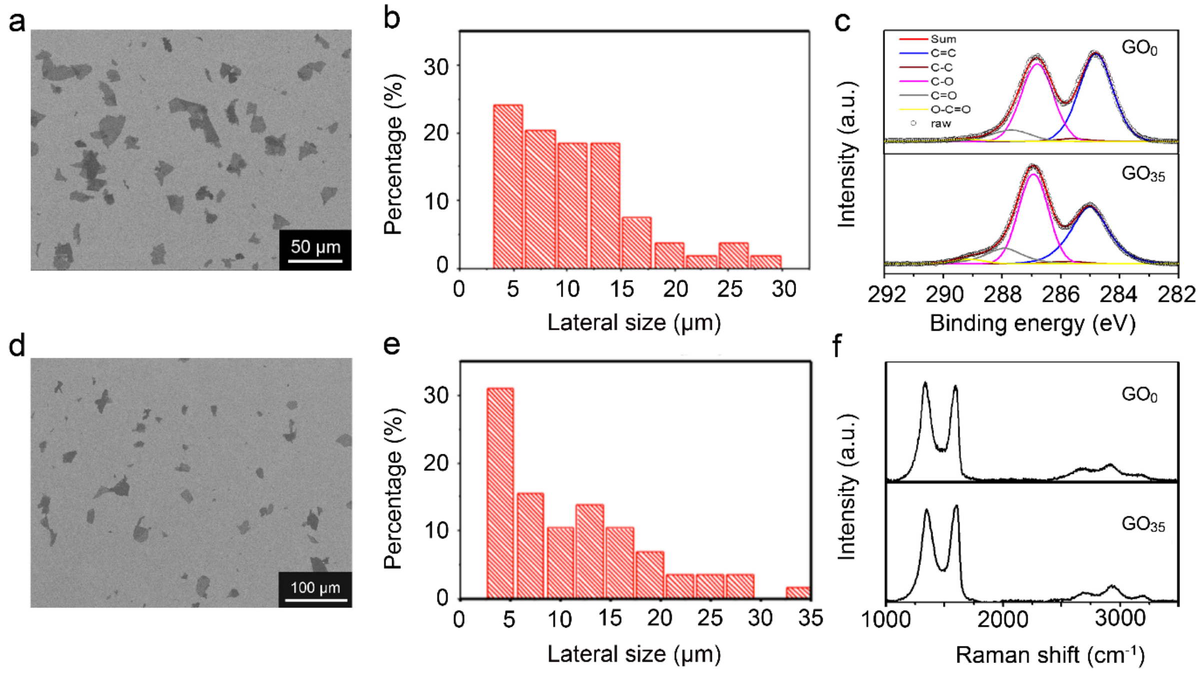

Supplementary Figure 1. Structural characterization of GO_0 and GO_{35} sheets. Scanning electron microscopy (SEM) images of GO_0 (**a**) and GO_{35} (**d**) sheets. The size-distribution histograms of GO_0 (**b**) and GO_{35} (**e**) measured from the SEM images. (**c, f**) XPS and Raman spectra of GO_0 and GO_{35} sheets. Source data are provided as a Source Data file.

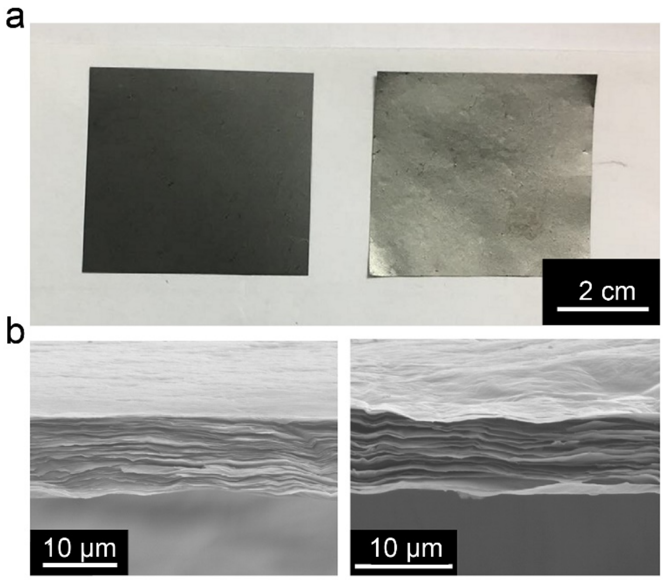

Supplementary Figure 2. CMG films and their SEM images. (a) Photographs of CMG (GO/left, rGO/right) films. Post-reduction of GO films gave rGO films with a metallic luster. (**b**) SEM cross-section images of the *e*-GO_0 (left) and *e*-rGO_0 (right) films, demonstrating the wavy textures of inter-connected network of CPLs in the CMG films.

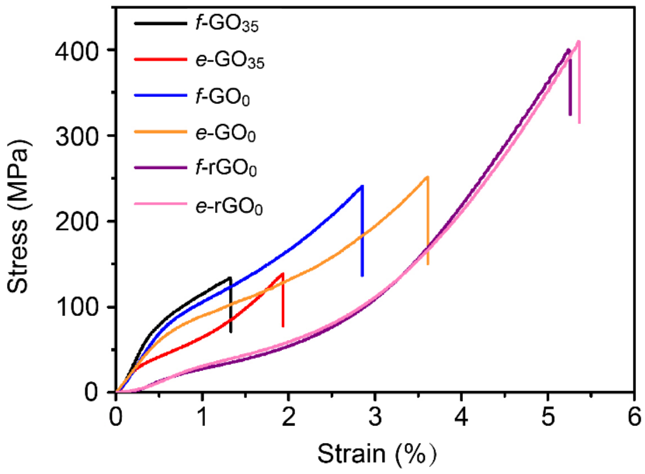

Supplementary Figure 3. Stress-strain curves of CMG films prepared from different GO precursors and film-forming methods. Source data are provided as a Source Data file.

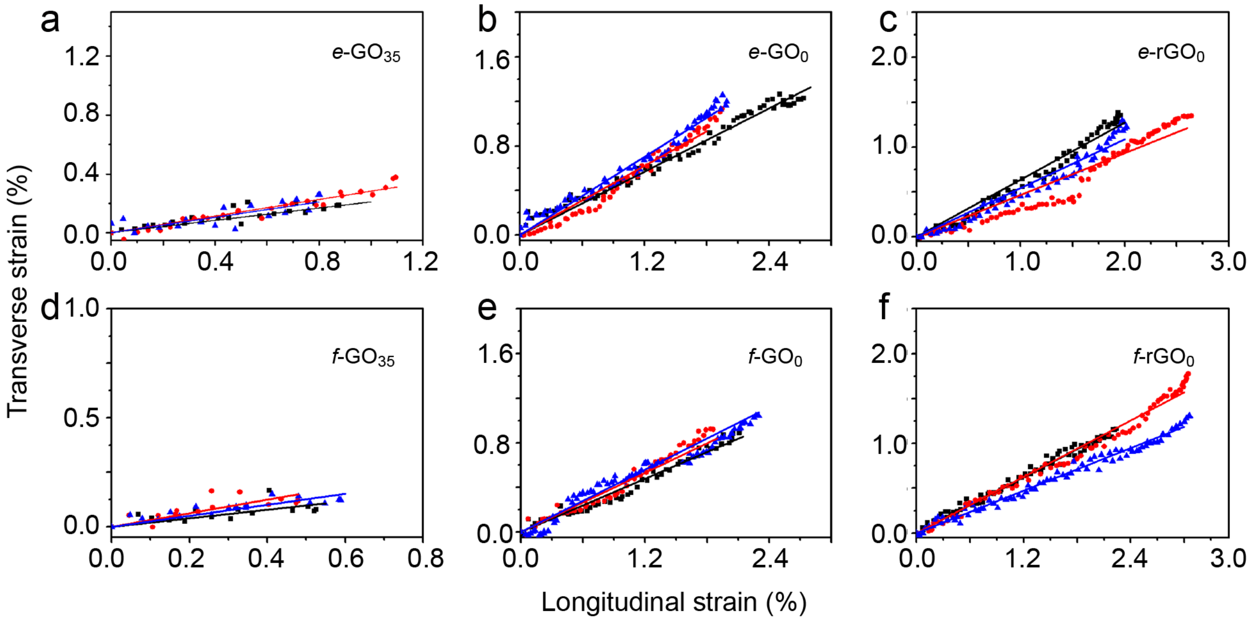

Supplementary Figure 4. Transverse strain plotted against the longitudinal strain applied to the CMG (GO or rGO) films. The average transverse strain of all samples (a, *e*-GO_{35}; b, *e*-GO_0; c, *e*-rGO_0; d, *f*-GO_{35}; e, *f*-GO_0; f, *f*-rGO_0) increases with the average longitudinal strain, demonstrating the NPR behavior. Source data are provided as a Source Data file.

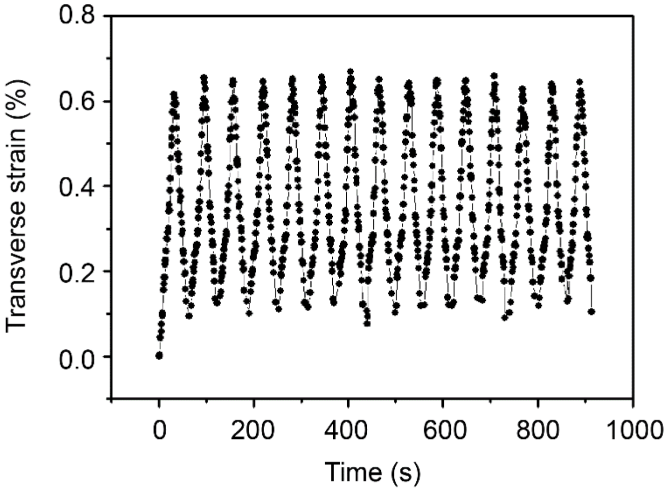

Supplementary Figure 5. Transverse strain plotted against time for *e*-GO_0 films under cyclic loading. The transverse strain is positive for the entire test, demonstrating the auxetic behavior of *e*-GO_0 films under cyclic loading-unloading tests with peak strain of 1.5%. Source data are provided as a Source Data file.

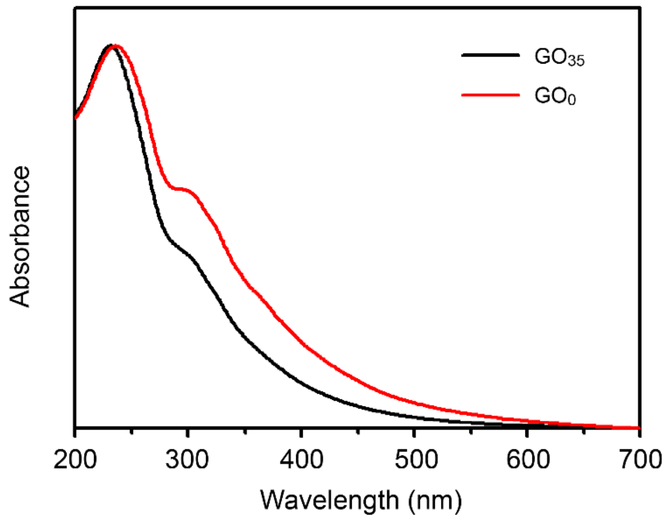

Supplementary Figure 6. UV-Vis absorption spectra of GO_0 and GO_{35} dispersions. The GO_0 sheets have a larger graphitic domain than that in GO_{35}. Source data are provided as a Source Data file.

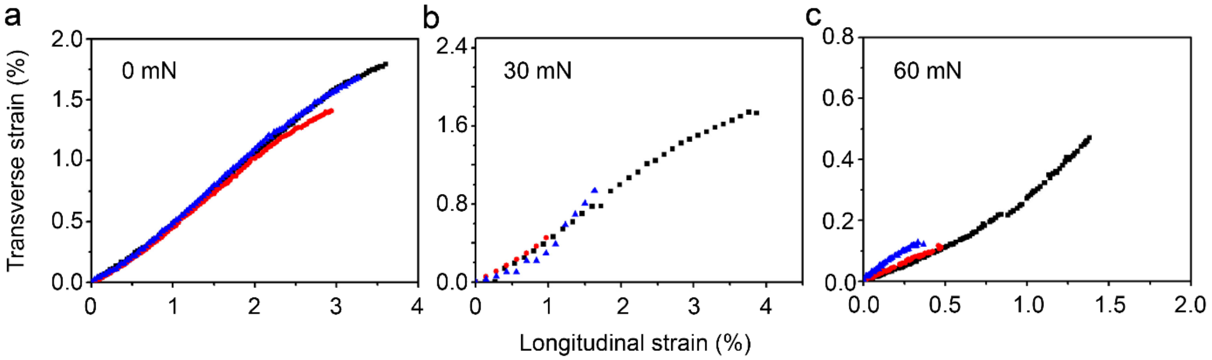

Supplementary Figure 7. Transverse strain plotted against the longitudinal strain for CMG films under pre-stretch of 0, 30 and 60 mN. The slope depends on the pre-stretch (0, 30 and 60 mN for panel a, b and c, respectively), indicating the tuned values of Poisson's ratios. Source data are provided as a Source Data file.

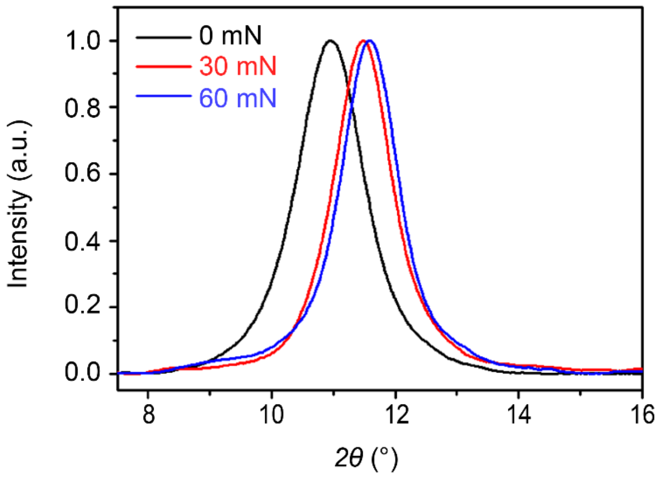

Supplementary Figure 8. XRD patterns of CMG films without and with pre-stretch (30 and 60 mN). Source data are provided as a Source Data file.

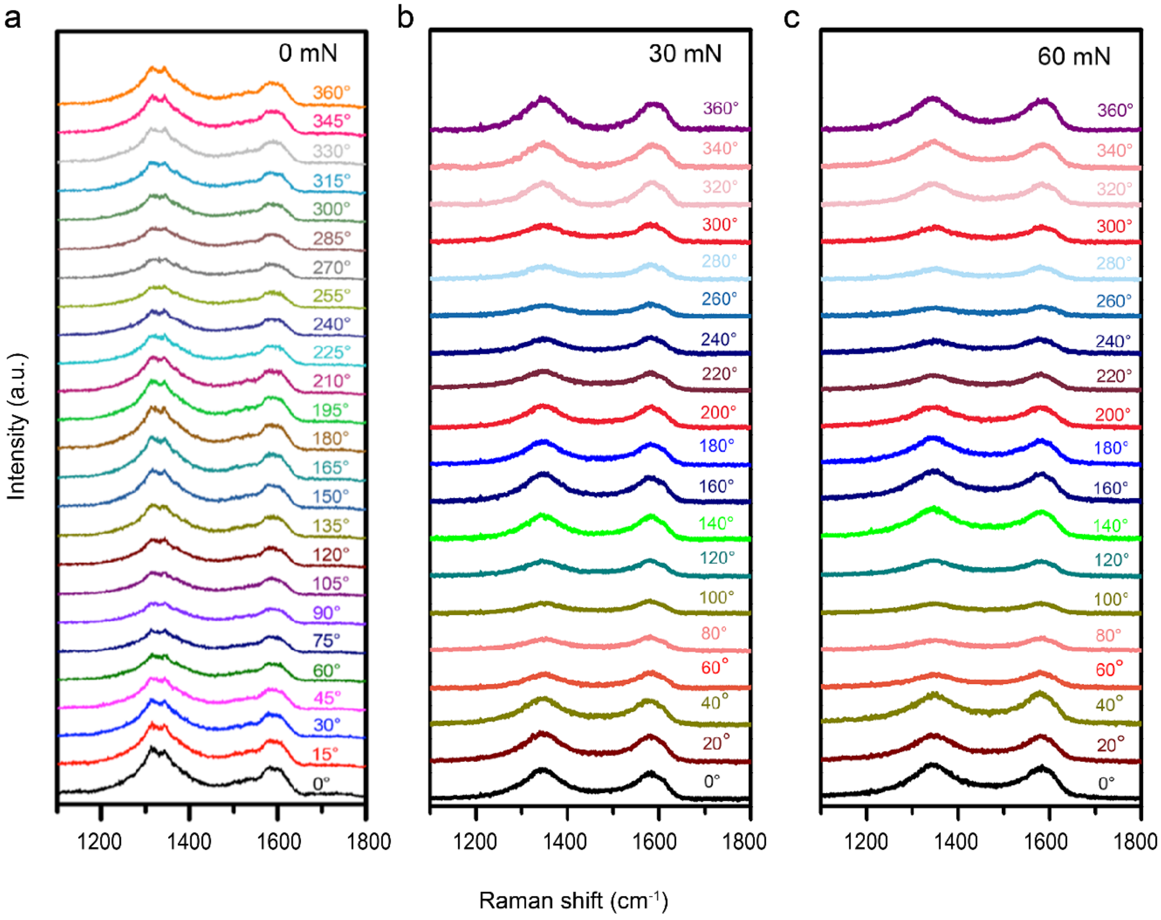

Supplementary Figure 9. Raman spectra for an *e*-GO$_0$ film under 0 (a), 30 (b) and 60 mN (c) pre-stretch. The *G*-band intensity in polarized Raman spectra of the *e*-GO$_0$ film changes with the angle β between the electric field vector of the incident laser and the base plane of the *e*-GO$_0$ film. Source data are provided as a Source Data file.
